# Rational Design and *in-situ* Synthesis of Ultra-Thin β-Ni(OH)_2_ Nanoplates for High Performance All-Solid-State Flexible Supercapacitors

**DOI:** 10.3389/fchem.2020.602322

**Published:** 2020-12-01

**Authors:** Shensong Wang, Changqin Tan, Linfeng Fei, Haitao Huang, Shujun Zhang, Hao Huang, Xinyi Zhang, Qiu-an Huang, Yongming Hu, Haoshuang Gu

**Affiliations:** ^1^Hubei Key Laboratory of Ferro- and Piezoelectric Materials and Devices, Faculty of Physics and Electronic Science, Hubei University, Wuhan, China; ^2^Department of Applied Physics, The Hong Kong Polytechnic University, Hong Kong, China; ^3^Institute for Superconducting & Electronic Materials, Australian Institute of Innovative Materials, University of Wollongong, Wollongong, NSW, Australia; ^4^College of Science/Institute for Sustainable Energy, Shanghai University, Shanghai, China

**Keywords:** β-Ni(OH)_2_, ultra-thin nanoplates, energy density, flexibility, all-solid-state supercapacitors

## Abstract

The all-solid-state flexible supercapacitor (AFSC), one of the most flourishing energy storage devices for portable and wearable electronics, attracts substantial attentions due to their high flexibility, compact size, improved safety, and environmental friendliness. Nevertheless, the current AFSCs usually show low energy density, which extremely hinders their practical applications. Herein, ultra-thin β-Ni(OH)_2_ nanoplates with thickness of 2.4 ± 0.2 nm are *in-situ* grown uniformly on Ni foam by one step hydrothermal treatment. Thanks to the ultra-thin nanostructure, β-Ni(OH)_2_ nanoplates shows a specific capacitance of 1,452 F g^−1^ at the scan rate of 3 mV s^−1^. In addition, the assembled asymmetric AFSC [Ni(OH)_2_//Activated carbon] shows a specific capacitance of 198 F g^−1^. It is worth noting that the energy density of the AFSC can reach 62 Wh kg^−1^ while keeping a high power density of 1.5 kW kg^−1^. Furthermore, the fabricated AFSCs exhibit satisfied fatigue behavior and excellent flexibility, and about 82 and 86% of the capacities were retained after 5,000 cycles and folding over 1,500 times, respectively. Two AFSC in series connection can drive the electronic watch and to run stably for 10 min under the bending conditions, showing a great potential for powering portable and wearable electronic devices.

## Highlights

- Ultra-thin β-Ni(OH)_2_ nanoplates with thickness of 2.4 nm are designed and *in-situ* synthesized.
- The all-solid-state flexible supercapacitors show a high energy density of 62 Wh kg^−1^.
- The supercapacitors exhibit excellent flexibility and stability and show great promise for practical applications.

## Introduction

With the rapid development of portable and wearable electronic devices, the demand for high-performance, flexible, and safe energy storage devices has increased dramatically energy (Huang et al., 2016). As a kind of emerging power sources, supercapacitors (SCs), whose power and densities are between the gap of batteries and dielectric capacitors, have drawn widespread attention owing to their ultrahigh power density, long lifetime, fast charging–discharging rate, etc. (Zhao et al., 2018). Compared to traditional liquid-electrolyte-based SCs, SCs based on solid-state electrolytes show their great potential for portable devices (Meng et al., 2010; Yang, 2020). However, up to now, the commercial application of AFSCs is still challenged because the energy density is much lower than that of rechargeable batteries. To date, a variety of materials have been explored as the electrode materials for fabricating high energy density SCs, which can be categorized into carbon materials and pseudocapacitive materials roughly (Peng et al., 2014). It has been demonstrated that pseudocapacitive materials are of great significance in achieving both high energy density and power density SCs such as transition metal oxides, hydroxides, dichalcogenides or nitrides, and conducting polymers (Wu et al., 2017). Among them, Ni(OH)_2_ has been extensively studied because of the fascinating features of excellent redox behavior, high theoretical capacity, and natural abundance. Especially, the unique two-dimensional (2D) laminar structure with sufficient interlayer space causing by intercalated water molecules and ions can provide transport channels for electrolyte and improve the charge storage ability (Zha et al., 2017). Generally, Ni(OH)_2_ has two types of crystal structures, i.e., α and β phase. In comparison to α-Ni(OH)_2_, β-Ni(OH)_2_ has better stability and longer lifetime in an alkaline electrolyte (Cai et al., 2004). Nevertheless, analogous to other pseudocapacitive materials, β-Ni(OH)_2_ often shows an inferior capacity in practical application, which may be caused by the small specific surface area. It has been reported that special designed nanostructures, such as nanoflowers (Zhao et al., 2016), nanospheres (Qin et al., 2018), nanoplates (Wang et al., 2010), and nanowires (Dong et al., 2014), are effective approaches to improve the energy storage capacity of β-Ni(OH)_2_, which exhibit large capacitance because of their abundant active sites benefiting from large surface. However, as a kind of typical 2D nanomaterial, the layers aggregation and stacking of β-Ni(OH)_2_ caused by hydrogen bond and electrostatic interactions may impede the ion transport and decrease the active sites, resulting in unsatisfactory capacitance and rate capability (Zha et al., 2017). Consequently, to avoid aggregation and stacking, *in-situ* growing ultra-thin β-Ni(OH)_2_ with few layers is significant to improve the electrochemical performance.

In this work, β-Ni(OH)_2_ nanoplates with ultra-thin thickness are *in-situ* grown on Ni foam by a facile hydrothermal process at low temperature. An asymmetric AFSC based on ultra-thin β-Ni(OH)_2_ nanoplates was fabricated, in which the β-Ni(OH)_2_, PAAK/KOH, activated carbon (AC), and Ni foam served as the positive electrode material, electrolyte, negative electrode material, and current collector, respectively. The power density, energy density, reliability with cycling, and folding are systematically investigated. Finally, the practical applications of the asymmetric AFSC are also presented.

## Materials and Methods

### Preparation and Characterizations of Ultra-Thin β-Ni(OH)_2_ Nanoplates

**Figure 1:**
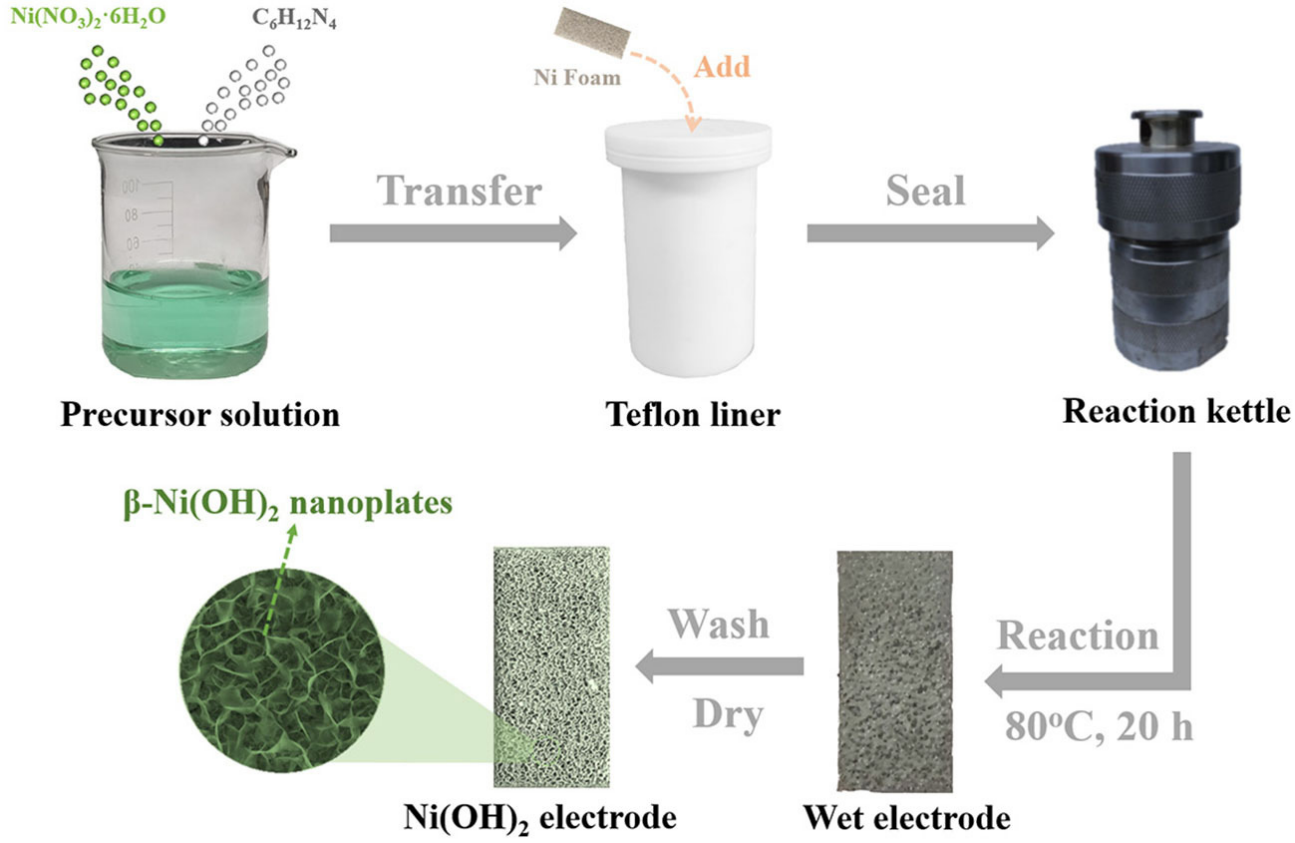
Schematic illustration of the procedure for the synthesis of ultra-thin β-Ni(OH)_2_ nanoplates.

All of the materials are of analytical grade and used without further purification. The preparation procedure is illustrated in Figure 1. Briefly, 1.1215 g of Ni(NO_3_)_2_·6H_2_O and 1.1632 g of C_6_H_12_N_4_ were added into a beaker containing 40 ml deionized water, and the mixture was magnetically stirred until it became clear to obtain the precursor solution. Then, a piece of Ni foam with size of 1 × 2 cm was cleaned by ultrasonication in acetone, 2 M HCl solution, and deionized water, respectively. After drying, the mass of Ni foam is marked m_0_. Subsequently, the clean Ni foam was added into a Teflon liner together with the precursor solution. After that, the Teflon liner was sealed by a reaction kettle and heated in an oven. After reaction at 80°C for 20 h, taking the Ni foam out and wash it with ethanol and deionized water, and then drying it at 60°C in vacuum. Finally, the color of Ni foam changed from the initial silvery white to light green after drying, and the mass was marked m_1_. The mass loading of β-Ni(OH)_2_ nanoplates (m) is about 1.6 mg cm^−2^, which can be calculated by equation: m = m_1_ – m_0_.

The structure, morphology, and composition of the samples were studied by using an X-ray diffractometer (XRD, D8 Advance, Bruker), a field-emission scanning electron microscope (FESEM; JEOL JSM7100F), and an atomic force microscope (AFM, Dimension FastScan, Bruker). X-ray photoelectron spectroscopy (XPS) measurement was performed on an ESCALAB 250xi XPS spectrometer, using monochromatic Al Ka X-rays.

### Assembly of Asymmetric AFSC and Electrochemical Measurement

The slurry of asymmetric AFSCs was prepared by mixing acetylene black, poly(vinylidene fluoride), and AC with N-methyl-2-pyrrolidone in a mass ratio of 1:1:8. Then, the slurry was dip-coated on the Ni foam (1 × 2 cm) and dried at 120°C for 10 h. The mass loading of negative material (AC) was calculated by Equation 1 based on the charge balance between positive and negative electrode (Guo et al., 2019).

(1) $$\begin{array}{l} \frac{m_{+}}{m_{-}}=\frac{C_{-}V_{-}}{C_{+}V_{+}}- \end{array}$$

Where *m*_+_ (g) and m_−_ (g) are the active material mass of positive and negative electrode, respectively. C_+_ (F g^−1^) and C_−_ (F g^−1^) stand for the specific capacitance of positive and negative electrode, respectively. *V*_+_ (V) and *V*_−_ (V) are the potential window of positive and negative electrode, respectively. The mass of the AC is about 1.6 mg cm^−2^, respectively.

By mixing 2 g of KOH, 2 g of PAAK, and 20 ml of deionized water under stirring at room temperature, the PAAK/KOH gel electrolyte is ready to use when it became clear and transparent. A filter paper was served as the separator. Finally, the three components [positive electrode β-Ni(OH)_2_, negative electrode AC, and separator] dip-coated with the prepared electrolyte were assembled into an asymmetric AFSC.

The electrochemical performance of the β-Ni(OH)_2_ electrode was studied using a three-electrode system. β-Ni(OH)_2_, Hg/HgO, platinum foil (2 m × 2 cm), and 1 M of KOH aqueous solution were used as the work electrode, reference electrode, counter electrode, and electrolyte, respectively. The electrochemical tests such as alternating current electrochemical impedance spectroscopy (EIS), galvanostatic charge/discharge (GCD), and cyclic voltammetry (CV) were performed by means of the Zahner electrochemical workstation (CIMPS-2). The cycling stability was studied by using the LANHE battery testing system (CT2001A). The electrochemical performance of the asymmetric AFSCs was also studied by the same testing devices.

## Results and Discussions

**Figure 2:**
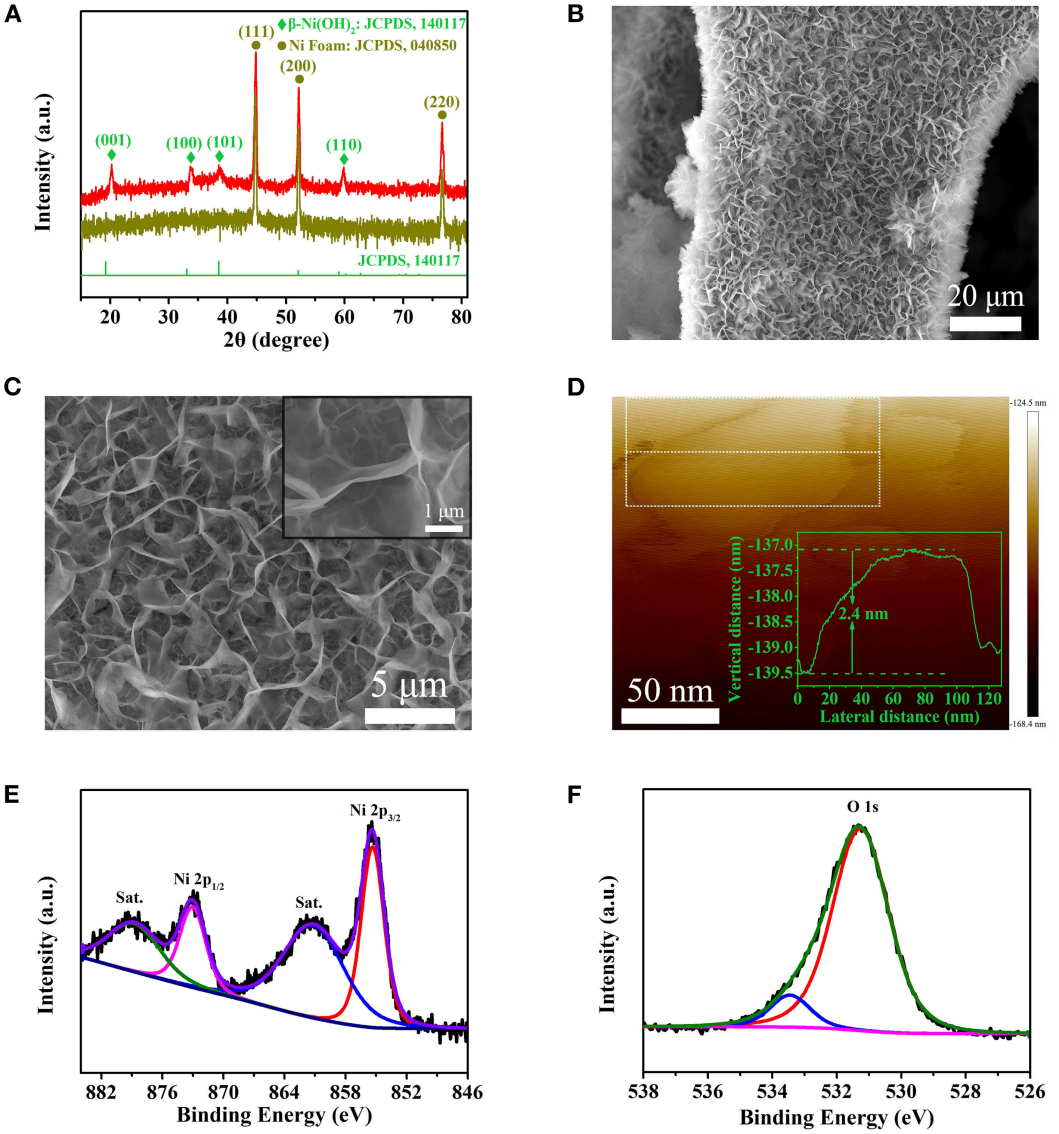
Phase, microstructural, and chemical state analysis of Ni(OH)_2_ nanoplates. **(A)** XRD patterns. **(B,C)** The SEM images at different magnifications. Inset of C is a high-magnification SEM image. **(D)** AFM image of ultra-thin Ni(OH)_2_ nanoplates, inset is the thickness curve. **(E)** XPS peaks of Ni 2p. **(F)** XPS peaks of O1s.

Figure 2A shows the XRD pattern of the as *in-situ* grown sample. In order to display the sample diffraction peaks more intuitively, the diffraction intensity has been processed by logarithm. One can see that the diffraction peaks at 20.2, 33.6, 38.8, and 59.8° correspond to (001), (100), (101), and (110) of Ni(OH)_2_, respectively, which is consistent with β-Ni(OH)_2_ (JCPDS 14-0117) (Ji et al., 2013). Meanwhile, there are other three dark yellow peaks locating at 44.5, 51.8, and 76.4°, which belong to the diffractions of the Ni foam (JCPDS 04-0850) (Kim et al., 2017). Figures 2B,C show the SEM images of β-Ni(OH)_2_ with different magnifications. It is obvious that the β-Ni(OH)_2_ nanoplates are *in-situ* grown uniformly on the surface of Ni foam, forming three-dimensional porous network structure. From the inset of Figure 2C, it can be seen that the nanoplates are extremely thin. In order to further determine the thickness of β-Ni(OH)_2_ nanoplates, AFM image is shown in Figure 2D and Supplementary Figure 1. It can be seen that the morphology is irregular after ultrasonic treatment. Using the Step Model in NanoScope Analysis Software, we can obtain the step height of selected area in nanoplate. Considering that there is a little error in the AFM test, the thickness distribution is shown in Supplementary Figure 1. It can be seen that the thickness of β-Ni(OH)_2_ nanoplate is about 2.4 ± 0.2 nm, which is much thinner than those of β-Ni(OH)_2_ in previous reports (Lu et al., 2011; Li et al., 2020). Figures 2E,F shows the XPS curves of Ni and O elements in Ni(OH)_2_ nanoplate. The peak of Ni 2p_3/2_ is located at 855.6 eV with a shake-up satellite (denoted as “Sat.”) peak at about 861.5 eV; meanwhile, Ni 2p_1/2_ characteristic peak is located at 873.2 eV with a shake-up satellite peak at about 879.9 eV. The spin-orbit splitting energy of Ni 2p1/2 and Ni 2p3/2 is at 17.6 eV, corresponding to the chemical state of Ni^2+^ (Chang et al., 2014). In addition, two oxygen bonds peaks can be observed from the O1s spectrum in Figure 2F. The peak at 531.2 eV is attributed to the nickel–oxygen bonds and the peak at 533.9 eV may be attributed to the physic/chemisorbed water on the surface of β-Ni(OH)_2_ nanoplates (Chang et al., 2014).

**Figure 3:**
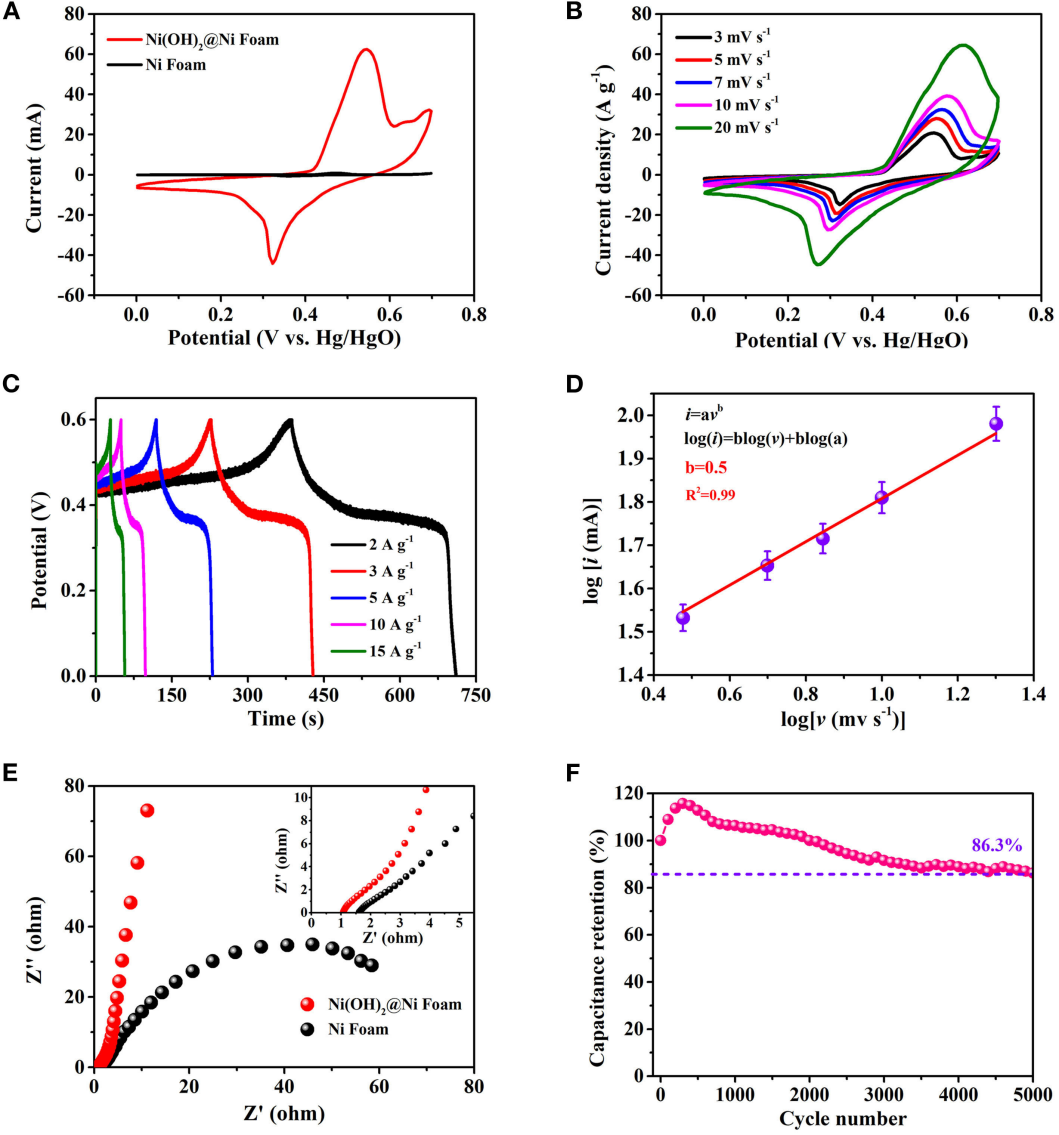
Three-electrodes electrochemical measurements of β-Ni(OH)_2_ electrodes in 1 M of KOH aqueous solution. **(A)** CV curves of the electrodes with scan rates from 3 to 20 mV s^−1^. **(B)** Specific capacitance and capacitance retention at different current densities. **(C)** GCD curves at various current densities. **(D)** Dependence of peak current and sweep speed after taking the logarithm. **(E)** Impedance Nyquist plots of the three-electrodes. **(F)** The cyclability of the electrode, the capacitance retention remains 86.3% after 5,000 cycles.

Figure 3 shows the electrochemical performance of as-prepared electrode based on ultra-thin β-Ni(OH)_2_ nanoplates [Ni(OH)_2_@Ni Foam] using the three-electrode system (vs. Hg/HgO). Figure 3A is the comparison of CV curves of bare Ni foam and Ni(OH)_2_@Ni Foam electrodes at a scan rate of 3 mV s^−1^. It can be seen that the curve of Ni Foam electrode has a quite small area, indicating that the Ni foam has a slight capacitance. In addition, after hydrothermal reaction, the Ni(OH)_2_ nanoplates grows on the surface of Ni foam, which may hinder the electrolyte from contacting the nickel foam extremely, resulting in a smaller capacitance contribution in energy storage. Supplementary Figure 2 shows the comparison of GCD curves, which also demonstrates that the Ni foam has a tiny capacitance. Therefore, we can approximately ignore the contribution of Ni foam in the capacitance calculation process. The CV curves of Ni(OH)_2_ nanoplates are obtained at different scan rates ranging in potential window from 0 to 0.7 V (Figure 3B). The typical irregular shapes with clear redox peaks can be observed, revealing that Faradaic redox reactions dominate the energy storage. The redox reaction during charging-discharging process might be described as follows:

(2) $$\begin{array}{l} \text{Ni}{\left(\text{OH}\right)}_{2}+\text{O}{\text{H}}^{-}⇌\text{NiOOH}+{\text{H}}_{2}\text{O}+e^{-} \end{array}$$

It's obvious that two redox peaks are almost symmetrical, indicating that the electrode based on β-Ni(OH)_2_ nanoplates has excellent reversibility. In addition, all the CV curves can still keep similar shape at different scan rates, revealing a good stability.

According to the CV curves, the specific capacitance (F g^−1^) of the β-Ni(OH)_2_ electrode can be calculated through following equation (Wu et al., 2017):

(3) $$\begin{array}{l} C=\frac{\oint I\text{dV}}{2mv\Delta V} \end{array}$$

Where *I* (A) is the instantaneous current in CV curve, *m* (g) is the mass of the active material, *v* (mV s^−1^) is the scan rate which represents the speed of the potential change during the positive and negative sweeps in the CV measurement, and Δ*V* (V) is the applied potential window which presents the range of potential change.

The specific capacitance and rate capability at different scan rates are shown in Supplementary Figure 3. Especially, the gravimetric capacitance achieves as high as 1,452 F g^−1^ at the scan rate of 3 mV s^−1^. Of particular importance is that β-Ni(OH)_2_ electrode also possesses excellent rate capability, remaining a superior capacitance value of 60% (869 F g^−1^) at a scan rate of 20 mV s^−1^, which attributes to the unique ultra-thin structure. Figure 3C shows the GCD curves obtained at different charging and discharging current densities. The obvious potential plateaus correspond to the pseudocapacitive behavior during the energy conversion and storage. Superior reversible redox capacity can also be demonstrated from the symmetric curves. To further understand the charge storage mechanism of as-prepared ultra-thin β-Ni(OH)_2_ nanoplates, the capacity contribution category is discussed in detail. According to the CV curves, the peak current *(i*, mA) and scan rate (*v*, mV s^−1^) obey the following functional relationship (Ju et al., 2020; Wang et al., 2020):

(4) $$\begin{array}{l} i=\text{a}{\text{v}}^{b} \end{array}$$

(5) $$\begin{array}{l} \text{log}\left(i\right)=\text{blog}\left(\text{v}\right)\;+\text{ blog}\left(\text{a}\right) \end{array}$$

Where a and b are adjustable parameters. After linear fitting log(*i*) and log(*v*), the curve shows in Figure 3D and the Adj. R-Square is about 0.99. According to the curve, it can be obtained that the *b*-value is 0.5, which means that the ultra-thin β-Ni(OH)_2_ nanoplates belongs to battery type material and the capacity comes from the Faraday intercalation reaction controlled by diffusion (Wang et al., 2007). This result corresponds to the GCD curve mentioned above, which has obvious potential plateaus as same as battery type material (Fleischmann et al., 2020). In this regard, designing ultra-thin nanostructure to avoid the layers from aggregation and stacking is of great significance. The *b*-values also indicate that the as-prepared β-Ni(OH)_2_ nanoplates have excellent channels for ions intercalation. As for the electrical and ionic conductivities of β-Ni(OH)_2_ electrode, the Nyquist plot with the frequency from 100 kHz to 10 mHz is obtained *via* the EIS-test (Figure 3E). In the low frequency region, the Nyquist plot is almost a vertical line, while the shape of bare Ni foam is like an semi-circle, suggesting the β-Ni(OH)_2_ nanoplates have good capacitive behavior. In the range of high frequency region, it can be concluded that the equivalent series resistance is about 1.1 Ω (R_ESR_, including the solution resistance, the contact resistance among active material and substrate, and the internal resistance of the active material) (Zhao et al., 2018), indicating a small electrode resistance as well as fast charge-transfer rate between the β-Ni(OH)_2_ nanoplates and electrolyte. Meanwhile, the bare Ni foam shows a larger R_ESR_ of 1.6 Ω, which may be due to that the nickel oxide formed on the surface of the Ni foam during charging and discharging hinders the charge transfer. The smaller R_ESR_ of Ni(OH)_2_ electrode also indicates that the Ni(OH)_2_ nanoplates growth on the surface of Ni foam can prevent electrolyte from contacting the nickel foam. Figure 3F shows the cycling stability of β-Ni(OH)_2_ electrode after charging-discharging 5,000 times at 3 A g^−1^. The capacitance increases about 15% during the first 300 cycles, which may be caused by the activation effect (Zhang et al., 2015). After 5,000 cycles, the specific capacitance stills retain 86.3% of the original value, demonstrating a good cycling stability.

**Figure 4:**
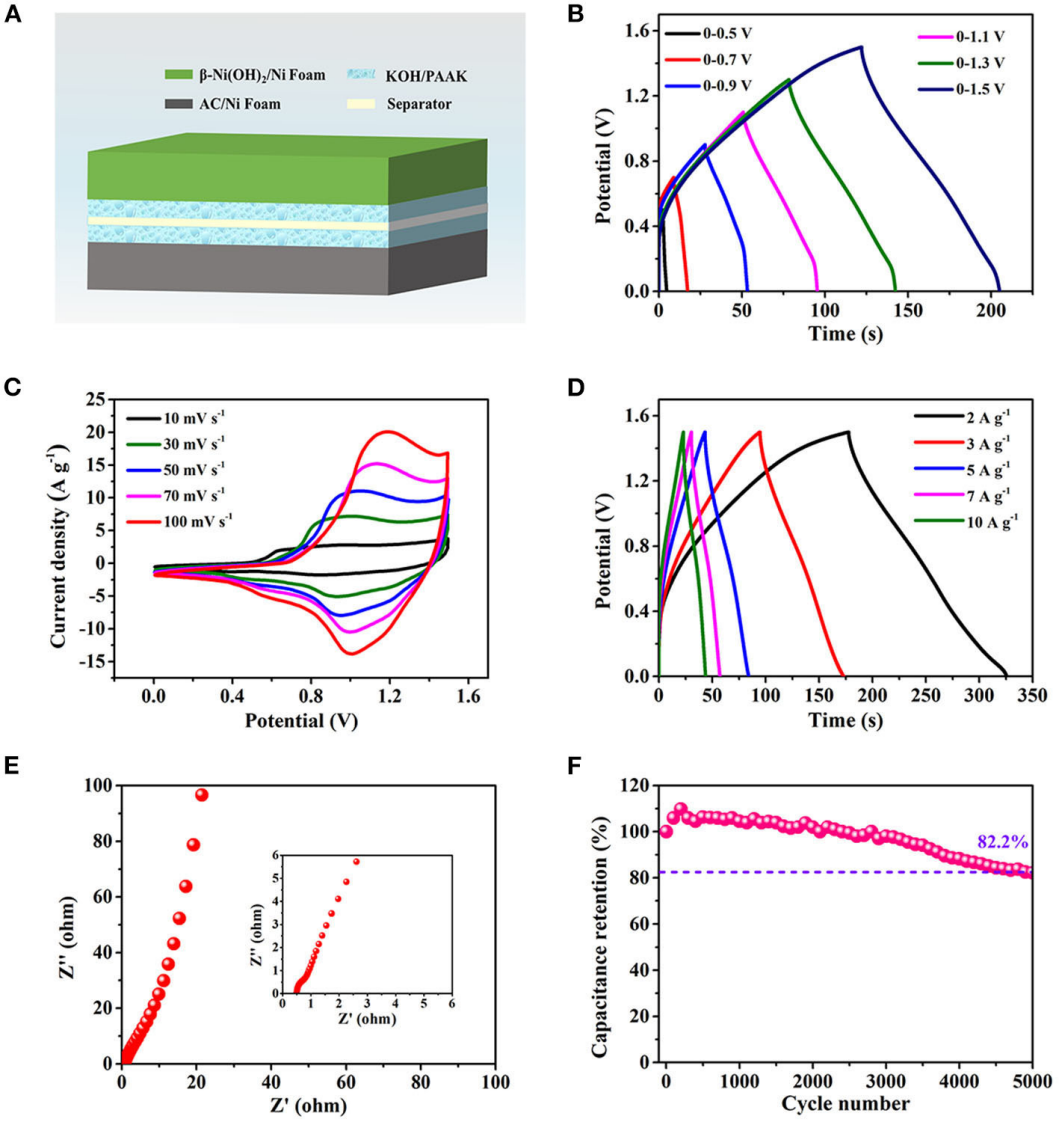
The electrochemical performance of the asymmetric AFSC device. **(A)** Schematic of the assembled AFSC device. **(B)** GCD curves at various potential windows. **(C)** CV curves at different scan rates. **(D)** GCD curves at different current densities. **(E)** EIS result of the AFSC. **(F)** Capacitance retention after 5,000 cycling times.

To further evaluate the application potential of β-Ni(OH)_2_ electrode for energy storage, a rechargeable asymmetric AFSC configuration is built based on AC negative electrode and β-Ni(OH)_2_ positive electrode, as schematically illustrated in Figure 4A. Figure 4B shows that the asymmetric AFSC has a high and stable potential window of 1.5 V, which is important to enhance the energy density. Figure 4C is the result of CV measurement for the assembled AFSC at different scan rates. There are obvious redox reactions during the charging and discharging process, and the similar shapes indicate the assembled AFSC has excellent stability. Figure 4D is the GCD curves at different current densities and the detail discharging curves are shown in Supplementary Figure 4.

According to the GCD curves, the specific capacitance (F g^−1^) of the AFSC can be calculated by Equation 6 (Wu et al., 2017):

(6) $$\begin{array}{l} C=\frac{I\Delta t}{m\Delta V} \end{array}$$

Where *I* (A) is the discharge current, *m* (g) is the mass of the active material, Δ*t* (s) is the discharge time, and Δ*V* (V) is the potential window.

The specific capacitances at different current densities are shown in Supplementary Figure 5. It can be seen that the capacitance is 198 F g^−1^, gradually decreasing to 104.6 F g^−1^ as the current density increasing from 2 to 15 A g^−1^, where a good capacity retention value of 53% is exhibited at 15 A g^−1^. This phenomenon maybe due to the diffusion movement of electrolyte ions is limited during high charging-discharging process. Thus, only the outer surface of β-Ni(OH)_2_ nanoplates can be used to storage charge, resulting in a low electrochemical utilization of the electrode materials.

To further study the capacitive behavior, the Nyquist plot is presented in Figure 4E. A straight line can be observed in the range of low frequency, the slope of which represents the Warburg resistance (Rw), which is caused by the diffusing resistance of electrolyte ions transferring into the interior of ultra-thin β-Ni(OH)_2_ nanoplates (Zhao et al., 2018). The straight line almost parallels to the imaginary axis, revealing the β-Ni(OH)_2_ electrode has low Rw and fast ions diffusion. From the magnified area of the high frequency portion, it can be determined that the R_ESR_ of the asymmetric AFSC is 0.5 Ω (Inset of Figure 4E). Moreover, the β-Ni(OH)_2_-based AFSC also shows good cycling stability (Figure 4F). After 5,000 cycles at 3 A g^−1^, the capacitance still retains 82.2%.

In addition, the energy density (E, Wh kg^−1^) and power density (P, W kg^−1^) of the AFSC can be calculated by the following equations (Wu et al., 2017):

(7) $$\begin{array}{l} E\;=\;\frac{C\Delta V^{2}\times 1000}{2\times 3600} \end{array}$$

(8) $$\begin{array}{l} P\;=\;\frac{E\times 3600}{\Delta t} \end{array}$$

Where C (F g^−1^) is the specific capacitance, Δ*V* (V) is the potential window and Δ*t* (s) is the discharge time.

**Figure 5:**
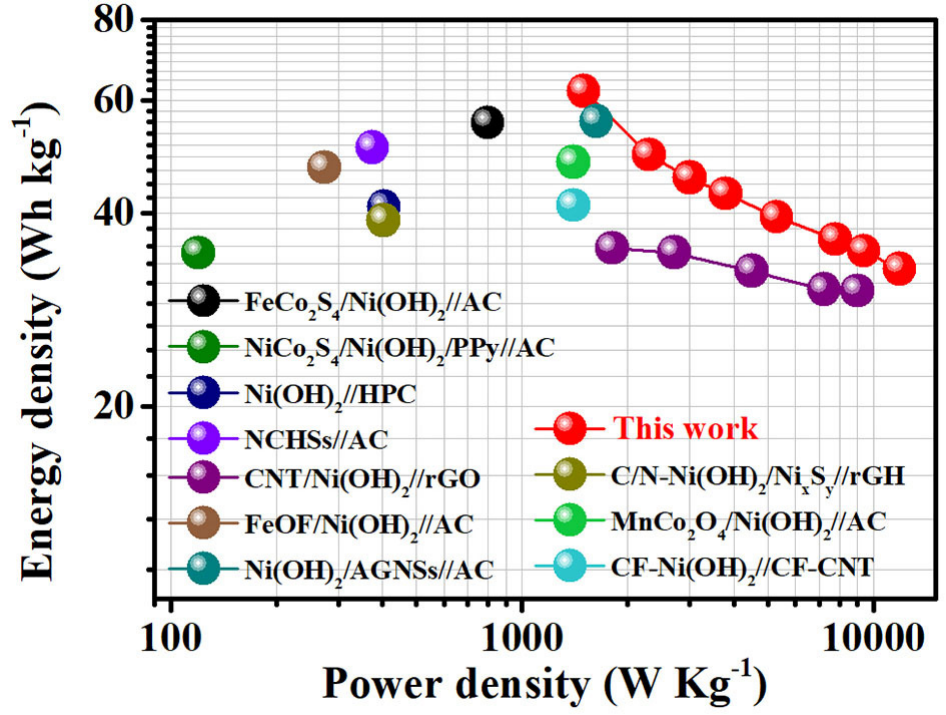
Power densities and energy densities of the AFSC devices in comparison with the state-of-the-art reported supercapacitors based on Ni(OH)_2_ materials.

Figure 5 shows the Ragone plot of performance comparison between our AFSC and the state-of-the-art reported supercapacitors based on Ni(OH)_2_. Impressively, at a power density of 1.5 kW kg^−1^, the AFSC can keep a high energy density of 62 Wh kg^−1^. Even under a high power density of 12 kW kg^−1^, the specific energy density can still achieve 32.7 Wh kg^−1^. Moreover, it is worth noting that the value reported here has exceeded those reported work recently such as FeCo_2_S_4_/Ni(OH)_2_//AC (55.3 Wh kg^−1^), NiCo_2_S_4_/Ni(OH)_2_/PPy//AC (34.7 Wh kg^−1^), Ni(OH)_2_//HPC (40.9 Wh kg^−1^) etc. (Supplementary Table 1; Ghosh et al., 2015; Salunkhe et al., 2015; Zhao et al., 2016; Wang et al., 2017; Liang et al., 2018; Liu et al., 2018; Qin et al., 2018; Yang et al., 2018; Zhang et al., 2018; Zhou et al., 2020). The excellent electrochemical performance of the asymmetric AFSC is attributed to the unique nanostructure of β-Ni(OH)_2_. The nanoplates *in-situ* growing on Ni Foam form a porous network structure, which can not only prevent aggregation but also improve ion transport in the whole electrode and the electrolyte accessibility of β-Ni(OH)_2_. Meanwhile, the ultra-thin thickness of the nanoplates can effectively avoid the influence caused by layers stacking. (All detailed electrochemical performance of the AFSC is listed in Supplementary Table 2.)

**Figure 6:**
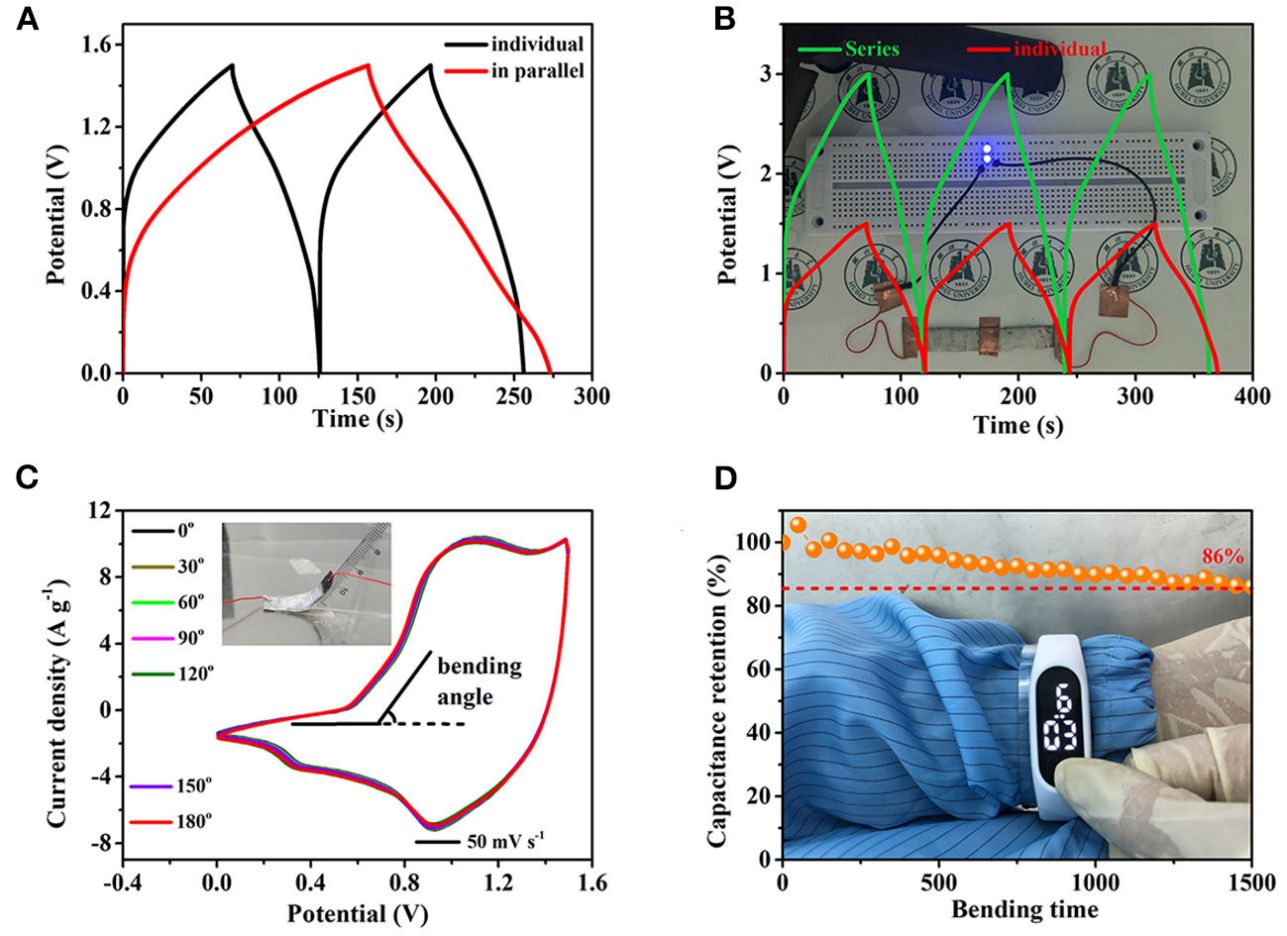
The practical application of AFSC devices. **(A)** GCD curves of two AFSC devices connected in parallel. **(B)** GCD curves of two AFSC devices connected in series, the background is the two working blue LEDs. **(C)** Bending angle-tests of the AFSC, inset is the digital photograph. **(D)** Capacitance retention of the AFSC after 1,500 times (bending angle: 180°), the background shows a digital watch driven by two AFSCs connected in series.

Considering that different applications have special requirements for capacity and potential, two AFSCs are assembled in both parallel (Figure 6A) and series connections (Figure 6B). Obviously, compared with the individual AFSC, the discharge time of two AFSC in parallel connection is much longer at the same current density, indicating that the capacity is increased by parallel connection. In the meantime, the potential window of two ASFCs connected in series is enlarged to 3 V. These results indicate that fabricated asymmetric AFSC can satisfy different demands in term of potential window and capacity. The background of Figure 6B is two blue LEDs in parallel, which can run for 5 min powered by two ASFCs connected in series charged to 3 V. Similarly, an electronic watch also can be driven by two AFSCs (Supplementary Figure 6). Flexibility is also an importance index for practical application. As shown in Figure 6C, at different bending angles, the changes in size and shape of the CV curves are almost unchanged, indicating that the AFSC still maintains good chemical stability under bending. Moreover, after folding for 1,500 times at 180 degrees, the capacity still maintains 86% of the original value, which further confirms the good flexibility of the AFSC (Figure 6D). Besides, attaching two AFSCs in series to the PET board and wrapping them around the hand, an electronic watch still can be powered stably for 10 min. These results give evidence that the fabricated AFSC possesses a high potential for applying in flexible and wearable electronic device.

## Conclusions

Ultra-thin β-Ni(OH)_2_ nanoplates grown on Ni foam are successfully prepared by a facile method. The β-Ni(OH)_2_ electrode exhibits a large specific capacitance and high rate capability. The asymmetric AFSC shows superior performance such as potential window (1.5 V), energy density (62 Wh kg^−1^ at the power density of 1.5 kW kg^−1^), cycling stability (about 82% capacitance remained over 5,000 cycles), and flexibility (about 86% capacitance remained over 1,500 folding times). The demonstrated performance for AFSCs suggests a great potential to convert and store energy for portable and wearable electronic devices. This work demonstrates that the rational design of ultra-thin nanostructure is an effective strategy to improve the electrochemical performance of 2D materials.

## Data Availability Statement

The raw data supporting the conclusions of this article will be made available by the authors, without undue reservation.

## Author Contributions

All authors listed have made a substantial, direct and intellectual contribution to the work, and approved it for publication.

## Conflict of Interest

The authors declare that the research was conducted in the absence of any commercial or financial relationships that could be construed as a potential conflict of interest.
