# Supplementary material for: Rational Design and in-situ Synthesis of Ultra-Thin β-Ni(OH)2 Nanoplates for High Performance All-Solid-State Flexible Supercapacitors
Source: Front Chem. 2020 Dec 1;8:602322. doi: 10.3389/fchem.2020.602322 (PMC7733587; doi:10.3389/fchem.2020.602322)
Supplement: Supplementary file 1 [file Table_1.DOC]

Supporting Information

**Rational Design and In-situ Synthesis of Ultra-thin β-Ni(OH)_2_ Nanoplates for High Performance All-Solid-State Flexible Supercapacitors**

Shensong Wang,^1^ Changqin Tan,^1^ Linfeng Fei,^3^ Haitao Huang,^3*^ Shujun Zhang,^4^ Hao Huang,^1^ Xinyi Zhang,^1^ Qiu-an Huang,^2^ Yongming Hu,^1*^ Haoshuang Gu^1^

*^1^ Hubei Key Laboratory of Ferro- & Piezoelectric Materials and Devices, Faculty of Physics and Electronic Science, Hubei University, Wuhan 430062, PR China*

*^2^ College of Science / Institute for Sustainable Energy, Shanghai University, Shanghai 200444, China*

*^3^ Department of Applied Physics, The Hong Kong Polytechnic University, Hong Kong, China*

*^4^ ISEM, Australian Institute of Innovative Materials, University of Wollongong, Wollongong, NSW 2500, Australia*

Corresponding Author: [huym@hubu.edu.cn](mailto:huym@hubu.edu.cn), [aphhuang@polyu.edu.hk](mailto:aphhuang@polyu.edu.hk).


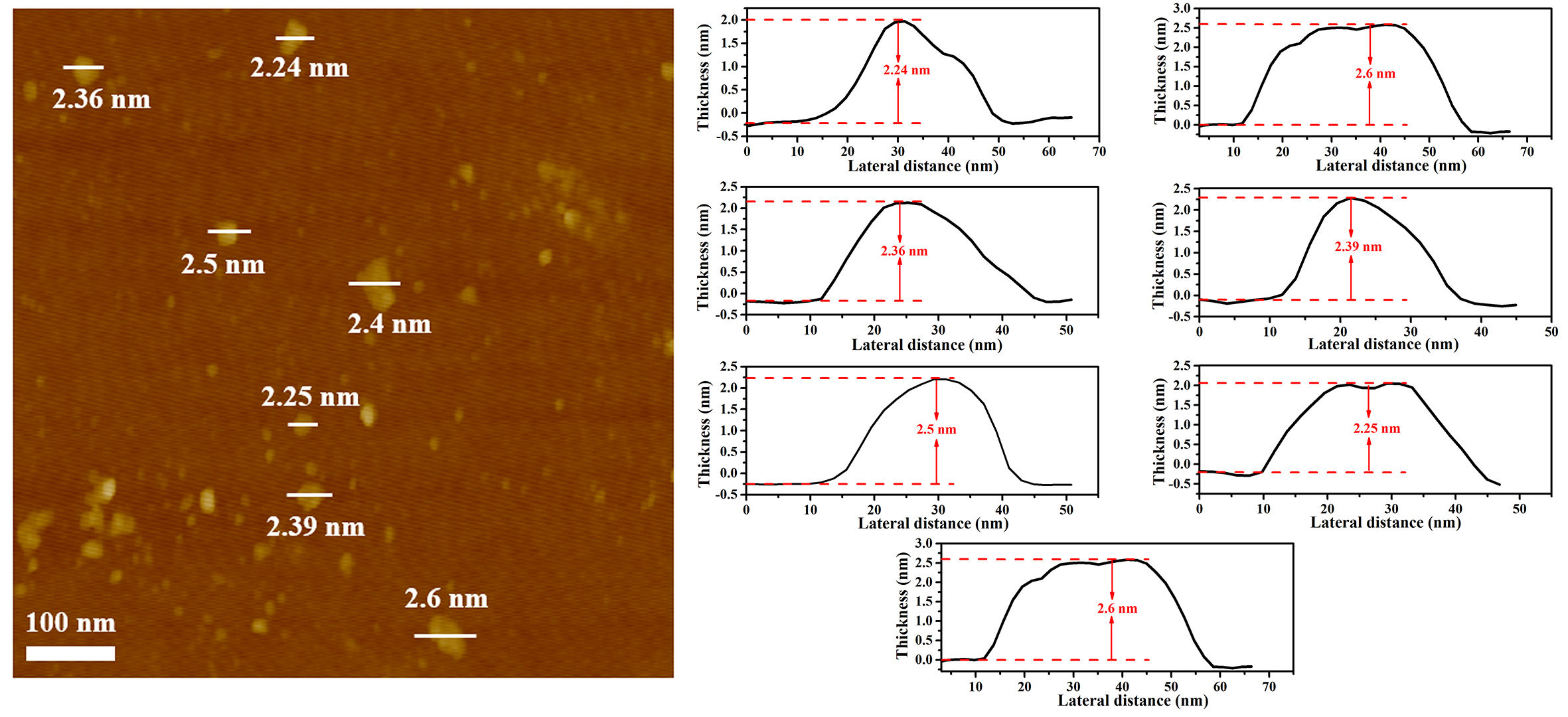


**Figure S1.** The thickness distribution of ultra-thin β-Ni(OH)_2_ nanoplates.


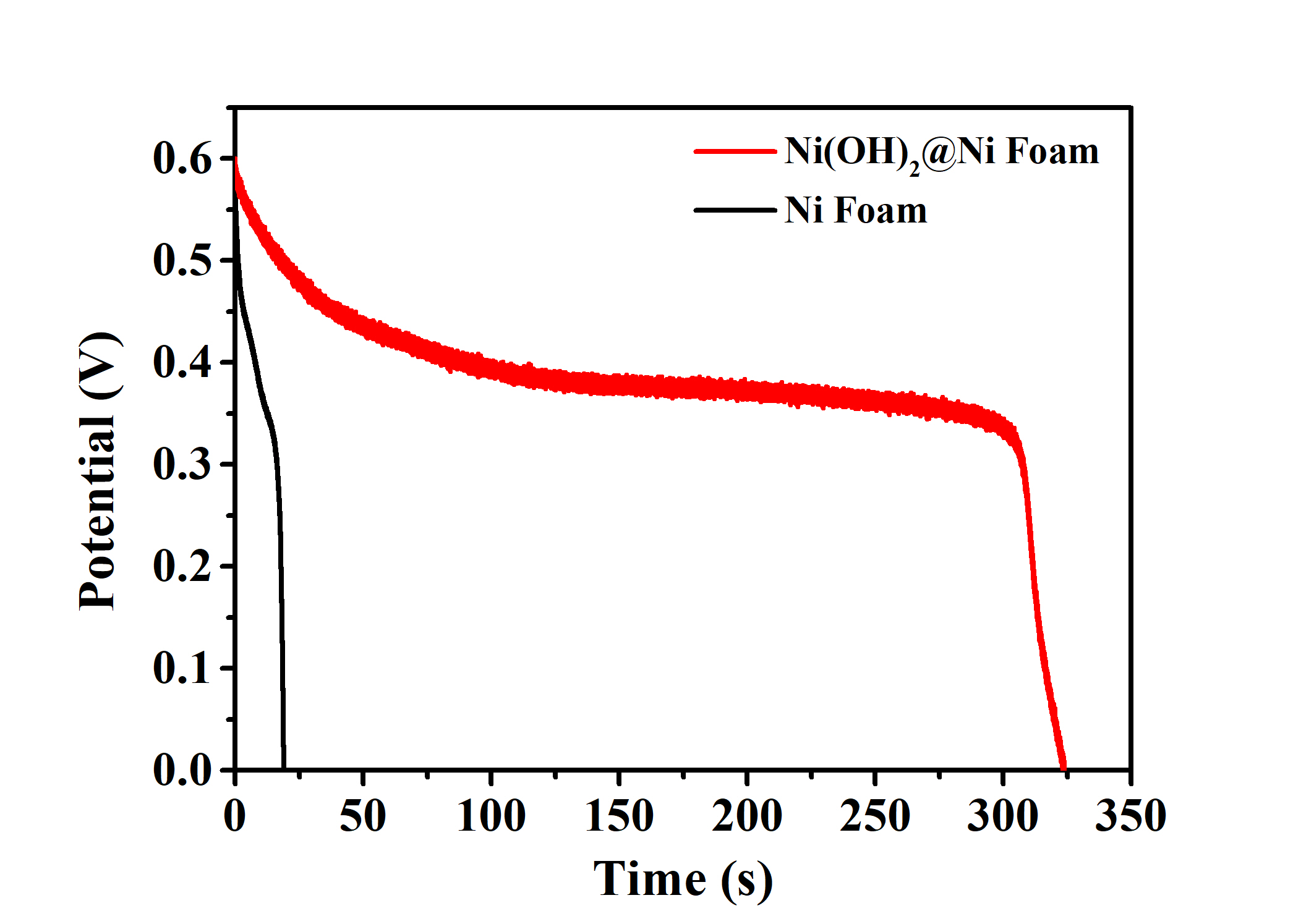


**Figure S2.** The discharging curves of Ni(OH)_2_@Ni Foam and Ni Foam at different current of 3.2 mA.


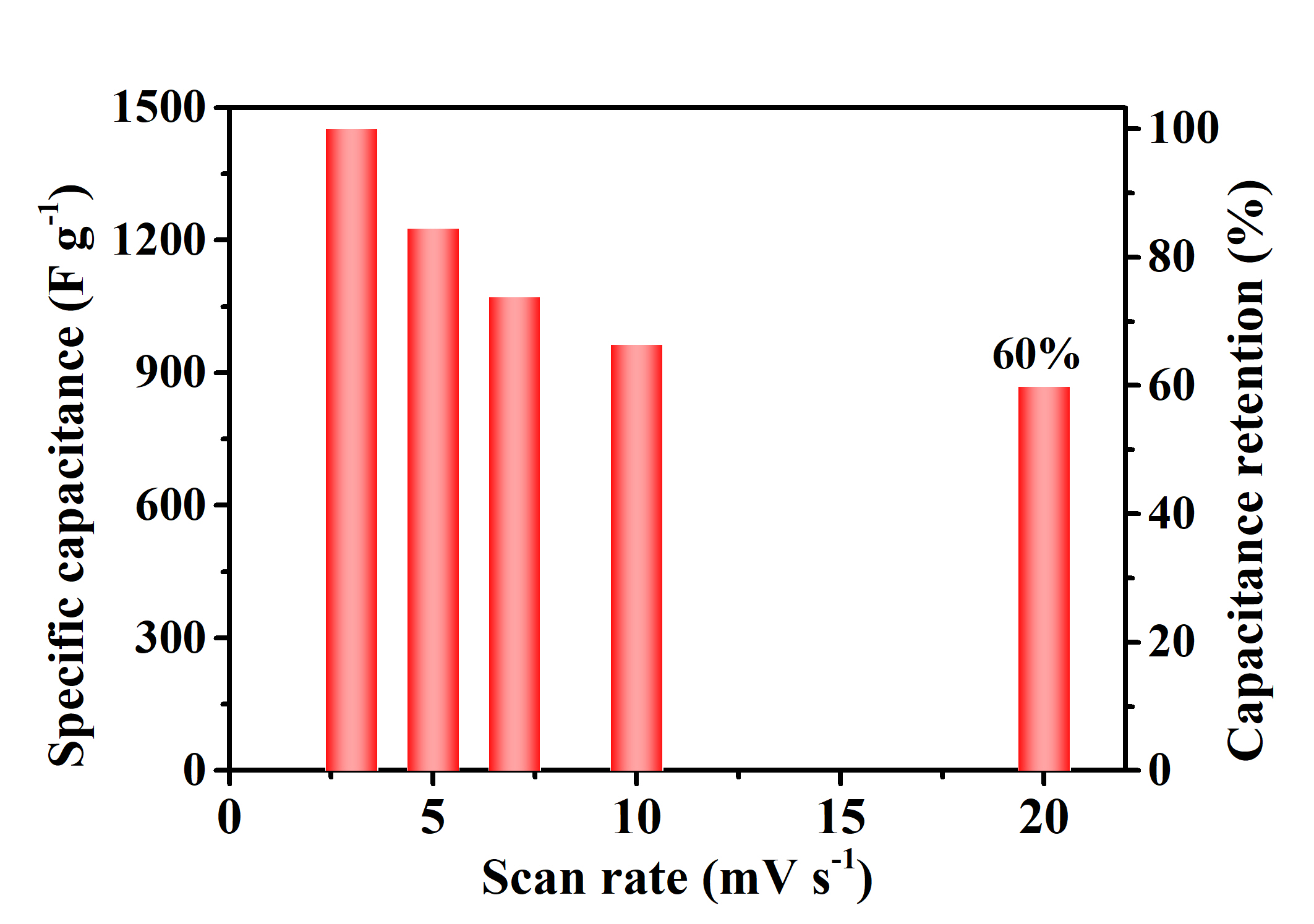


**Figure S3.** The specific capacitance and capacitance retention of Ni(OH)_2_ nanoplates at different current densities


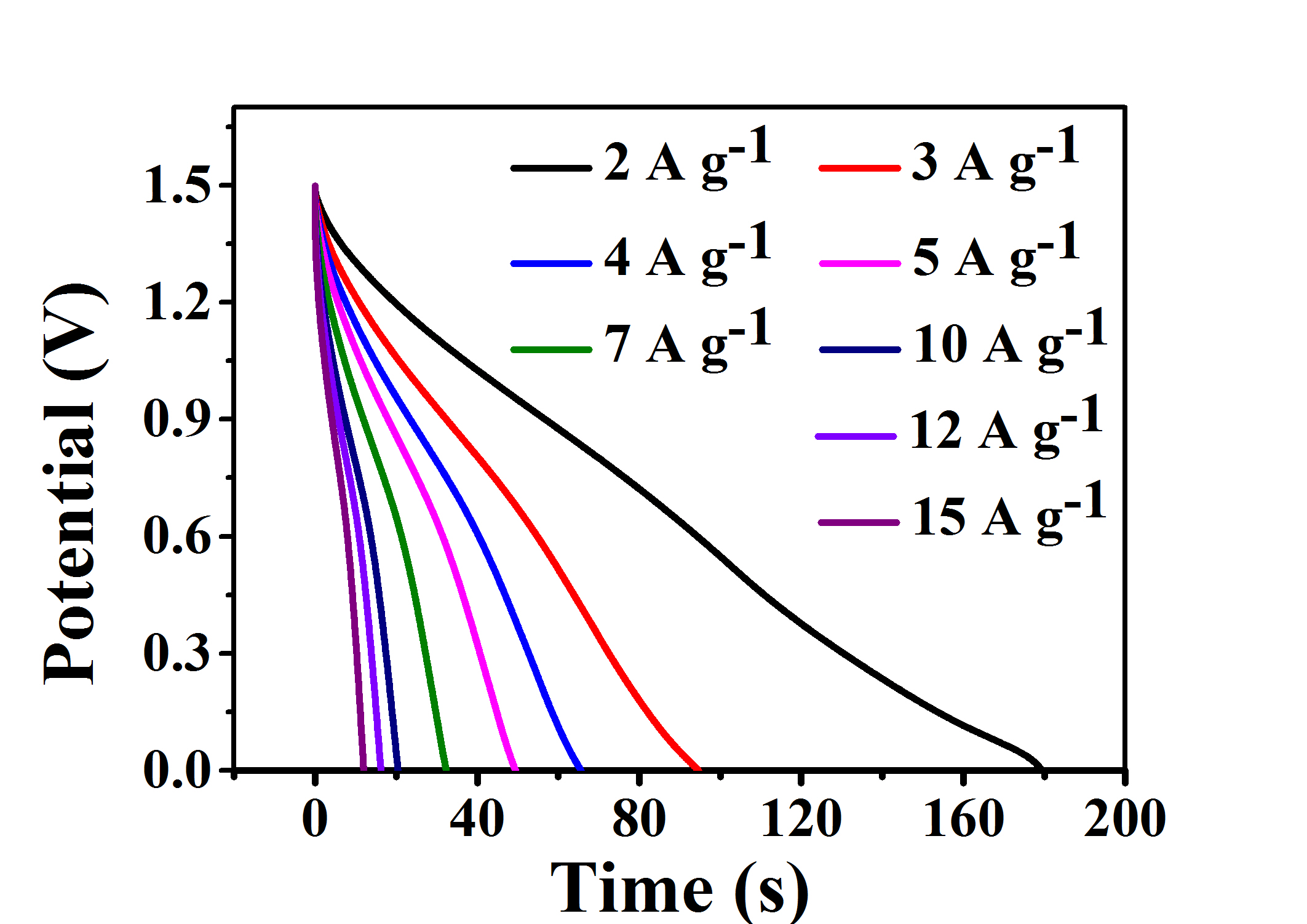


**Figure S4.** The discharging curves of Ni(OH)_2_//AC supercapacitors at different current densities.


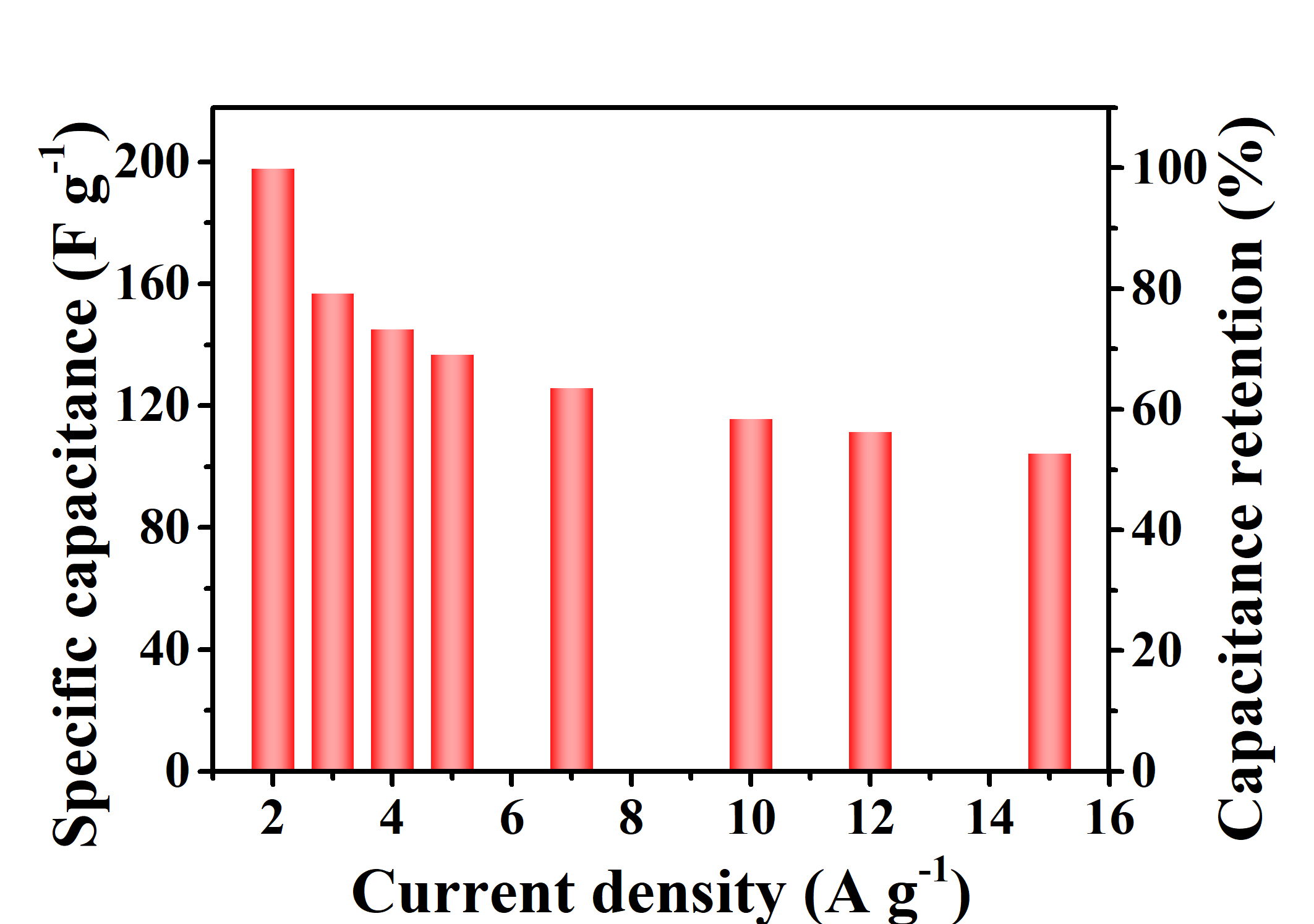


**Figure S5.** Specific capacitance of Ni(OH)_2_//AC supercapacitors at different current densities.

**Table S1.** The specific capacitance and energy density comparison of supercapacitors based on Ni(OH)_2_ materials

| **Electrode** | **Electrolyte** | **Specific capacitance** | **Energy density** |
| --- | --- | --- | --- |
| **NiCo_2_S_4_@Ni(OH)_2_@PPy//AC** | 2 M KOH | 1.983 F cm^-2^ at 5 mA cm^-2^ | 34.67 Wh kg^-1^ at a power  density of 120.127 W kg^-1^ |
| **Ni(OH)_2_//HPC** | 6 M KOH | 115 F g^-1^ at 0.5 A g^-1^ | 40.9 Wh kg^-1^ at a power  density of 405 W kg^-1^ |
| **3D NCHSs//AC** | PBI/KOH | 162 F g-^1^ at 0.5 Ag^-1^ | 50.6 Wh kg^-1^ at a power  density of 375 W kg^-1^ |
| **CNTNi(OH)_2_//rGO** | 1 M KOH | 78.3 F g^-1^ at 1 A g^-1^ | 35.24 Wh kg^-1^ at a power  density of 1.8 kW kg^-1^ |
| **FeOF@Ni(OH)_2_//AC** | PVA/KOH | 100.6 F g^-1^ at 1 A g^-1^ | 47.1 W h kg^−1^ at a power  density of 274 W kg^−1^ |
| **Ni(OH)_2_@AGNSs//AC** | 6 M KOH | 156.3 F g^-1^ at 2 A g^-1^ | 55.6 Wh kg^-1^ at a power  density of 1.628 kW kg^-1^ |
| **C/N-Ni(OH)_2_/NixSy//rGH** | 1 M KOH | --- | 38.98 Wh kg^-1^ at a power  density of 404.36 W kg^-1^ |
| **MnCo_2_O_4_@Ni(OH)_2_//AC** | 2 M KOH | 141 F g^-1^ at 0.5 A g^-1^ | 48 Wh kg^-1^ at a power  density of 1.4 kW kg^-1^ |
| **Mg-Ni(OH)_2_//AC** | 6 M KOH | 167 F g^-1^ at 1 A g^-1^ | 57.9 Wh/kg at a power  density of 1.58 kW kg^-1^ |
| **CF-Ni(OH)_2_// CF-CNT** | PVA/KOH | 151 F g^-1^ at 2 A g^-1^ | 41.1 Wh kg^-1^ at a power  density of 1.4 kW kg^-1^ |
| **Ni(OH)_2_//AC** | **PVA/KOH** | **240 F g^-1^ at 2 A g^-1^** | **75 Wh kg^-1^ at a power**  **density of 1.5 kW kg^-1^** |

**Table S2.** Performance data for the β-Ni(OH)_2_//AC Supercapacitors

| **Current density (A g^-1^)** | **Specific capacitance**  **(F g^-1^)** | **Energy density (Wh kg^-1^)** | **Power density (kW kg^-1^)** |
| --- | --- | --- | --- |
| **2** | **198** | **62** | **1.5** |
| **3** | **157** | **49.3** | **2.3** |
| **4** | **145.2** | **45.4** | **3** |
| **5** | **137** | **42.9** | **3.8** |
| **7** | **126** | **39.4** | **5.3** |
| **10** | **115.8** | **36.4** | **7.8** |
| **12** | **111.7** | **34.9** | **9.4** |
| **15** | **104.6** | **32.7** | **11.9** |


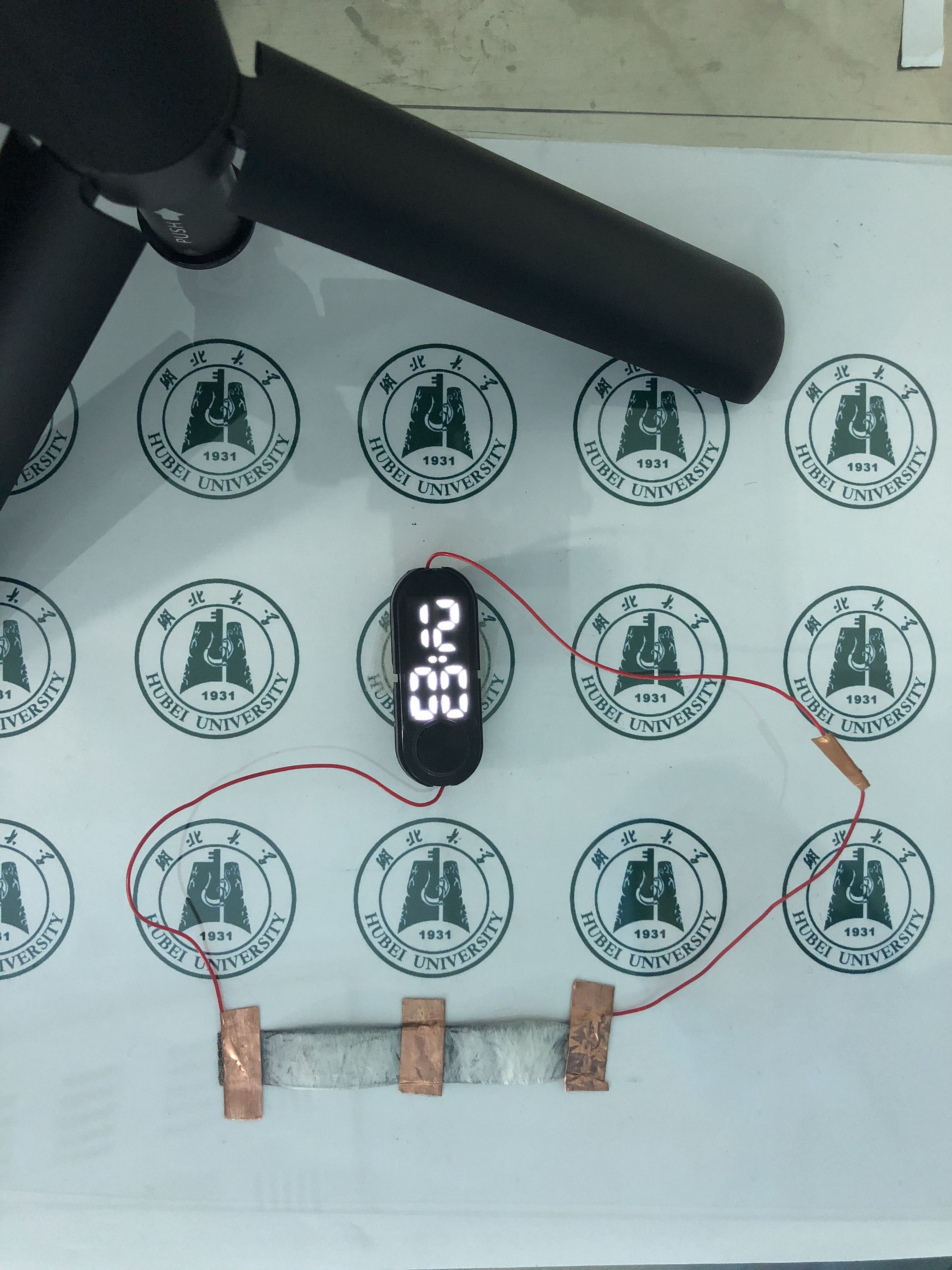


**Fig. S6.** Digital photograph shows an electronic watch driven by two devices in series connection.
